# Interpretation of a 12-Lead Electrocardiogram by Medical Students: Quantitative Eye-Tracking Approach

**DOI:** 10.2196/26675

**Published:** 2021-10-14

**Authors:** Mohammed Tahri Sqalli, Dena Al-Thani, Mohamed B Elshazly, ‪Mohammed Al-Hijji

**Affiliations:** 1 Information and Computing Technology Division College of Science and Engineering Hamad Bin Khalifa University Doha Qatar; 2 Weill Cornell Medical College Cornell University Doha Qatar; 3 Department of Cardiac Electrophysiology Cleveland Clinic Cleveland, OH United States; 4 Interventional & Structural Cardiology Division Hamad Medical Corporation Doha Qatar

**Keywords:** eye tracking, electrocardiogram, ECG interpretation, medical education, human-computer interaction, medical student, eye, tracking, interpretation, ECG

## Abstract

**Background:**

Accurate interpretation of a 12-lead electrocardiogram (ECG) demands high levels of skill and expertise. Early training in medical school plays an important role in building the ECG interpretation skill. Thus, understanding how medical students perform the task of interpretation is important for improving this skill.

**Objective:**

We aimed to use eye tracking as a tool to research how eye fixation can be used to gain a deeper understanding of how medical students interpret ECGs.

**Methods:**

In total, 16 medical students were recruited to interpret 10 different ECGs each. Their eye movements were recorded using an eye tracker. Fixation heatmaps of where the students looked were generated from the collected data set. Statistical analysis was conducted on the fixation count and duration using the Mann-Whitney U test and the Kruskal-Wallis test.

**Results:**

The average percentage of correct interpretations was 55.63%, with an SD of 4.63%. After analyzing the average fixation duration, we found that medical students study the three lower leads (rhythm strips) the most using a top-down approach: lead II (mean=2727 ms, SD=456), followed by leads V1 (mean=1476 ms, SD=320) and V5 (mean=1301 ms, SD=236). We also found that medical students develop a personal system of interpretation that adapts to the nature and complexity of the diagnosis. In addition, we found that medical students consider some leads as their guiding point toward finding a hint leading to the correct interpretation.

**Conclusions:**

The use of eye tracking successfully provides a quantitative explanation of how medical students learn to interpret a 12-lead ECG.

## Introduction

### Background

The electrocardiogram (ECG) is a graph that represents the electrical activity of the heart. The 12-lead ECG showcases this activity from 12 different “viewpoints” called leads. Although more than 300 million ECG tests are performed annually in the United States [1], relatively little is known about how these ECGs are interpreted among medical professionals. Despite the fact that an ECG is a standardized medical test, procedures and processes to interpret it significantly vary from country to country, let alone from one medical institution to another [2]. Several professional organizations, including the American Heart Association (AHA), the American College of Cardiology (ACC), and the Heart Rhythm Society (HRS), proposed recommendations for the standardization and interpretation of the ECG in 2007 [3]. The aim behind these recommendations was to “establish standards that will improve the accuracy and usefulness of the ECG in practice.” However, despite efforts of standardization, there still exists a variation amongst health care practitioners in their ability to accurately and quickly read ECGs. Understanding the origins of these differences in interpretation is important for improving and correcting training practices. Among the causes of this variation in the accuracy of ECG interpretation is how medical students are trained to perform the interpretation task while in medical school [4,5]. Understanding the chasm between how medical students are trained for ECG interpretation versus how they actually perform the task after receiving their medical training is important for improving and correcting training practices in the medical curriculum.

We present a quantitative study of ECG interpretation by 16 medical students. We requested from them to interpret 10 ECGs each while we collected their eye-tracking data using the eye-tracking methodology. We based our study on related works that looked at similar behavior in the general medical population. We sought to find a confirmation of our hypothesis that the order and the duration with which medical students look at specific areas across the ECG directly affect their final interpretation. We based our data analysis on understanding ECG interpretation within medical students using a number of different features, mainly areas of interest (AOIs), fixation count, fixation duration, time to first fixation (TTFF), and fixation revisitations. The results of the study uncover ECG interpretation insights among the population of medical students that confirm our hypothesis.

### Related Works

When eye tracking was first used to understand ECG interpretation, the aim was to gain insight into experts’ interpretation procedures. Bond et al [6] noticed that emerging trends in training practices delivered through medical schools and training centers seem especially tedious, rigorous and time-consuming [6]. Yet medical professionals find alternative ways of interpretation to keep up with the fast-running pace of the medical setting driven by detecting abnormalities rather than systematic interpretation of an ECG [7]. Thus, Bond et al [6] adopted eye tracking in order to understand how the collected eye gaze data may be used in order to unveil insights into how expert annotators proceed with ECG interpretation. Bond et al [6] collected the eye gaze data of several expert ECG interpretations. This was done with annotators viewing different ECGs each across different studies [4,6,8]. The authors then applied an objective quantitative approach to analyze the data. Bond et al’s [4,6,8] major findings were in quantifying the fixation duration over each lead of a 12-lead ECG. This provided an opportunity to compare the time spent fixating over leads in the frontal plane compared with leads in the transverse plane. Bond et al [4,6,8] also succeeded in ranking the leads that are looked at the most versus the least-looked-at leads. Bond et al’s [4,6,8] studies were insightful in facilitating the quantification of experts’ interpretation of an ECG.

However, a quantitative study alone might not be enough to unveil complex visual behaviors, such as ECG interpretation. Davies et al [2,5,7,9] therefore proposed additional studies that used mixed methods, combining both quantitative and qualitative components. Although the authors followed a similar eye-tracking study design as Bond et al [6], they improved their analysis by adding some quantitative and qualitative methods to better understand the eye-tracking data, such as computing the difference between eye transitions across the ECG. For the qualitative component of the study, however, Davies et al [2,5,7,9] aimed at better understanding the applied cognitive processes that expert interpreters refer to when interpreting ECGs. The authors interviewed and surveyed a set of medical practitioners. The authors found that medical experts develop their own style and system to quickly and efficiently interpret ECGs. Moreover, Davies et al [2,5,7,9] found that expert interpreters switch between their personally developed style of interpretation and a systematic method of interpretation, depending on the ECG and the severity of the symptoms it conveys.

Other studies, such as the studies done by Wood et al [10] and Breen et al [4], investigated eye-tracking interpretation of novice medical students as opposed to cardiology experts. The experiments resulted in quantifying the average time required by this sample of medical students to reach a final ECG interpretation. The results of Breen et al’s [4] study indicated that health care science students require between 13.2 and 59.5 seconds before reaching a final interpretation. Health care students rely mainly on lead II, as it was fixated on the longest. Wood et al [10] aimed through their study to understand the perceptual-cognitive mechanisms giving expert ECG interpreters an advantage over their novice counterparts. The authors found that expert interpreters are significantly faster, more accurate, and more confident in giving correct diagnoses. This difference in the accuracy of interpretation was also reflected upon the eye movement behavior of the consultants.

### Research Questions and Hypothesis

We asked our research questions based on the reviewed body of work. We then formed our hypothesis accordingly. We based our hypothesis on the results of previous related studies, as well as on our experience in teaching ECG interpretation to medical students.

#### Research Questions

Research question 1: *Can we recognize any eye-tracking patterns for ECG interpretation within the medical student population?*

Identifying patterns that medical students follow to interpret an ECG may explain the correct/incorrect interpretations they provide. Bond et al [6] observed that the majority of misinterpretations within expert interpreters are due to practitioners adopting only an initial first impression or pattern recognition approach to the ECG, without following a conventional systematic protocol to ECG interpretation.

In their study, Bond et al [6] used the accuracy of interpretation, the average fixation duration per lead, the average duration of individual fixations, and the TTFF as eye-tracking parameters to uncover the methodology followed by expert interpreters. We used similar parameters to quantify ECG interpretation behavior within the medical student population.

Research question 2: *How does the presence of waveform abnormalities across the ECG affect the eye movement behavior within medical students’ interpretation? Does it also affect their final diagnosis?*

Davies et al [5] observed that alterations in the normal shape of the ECG waveform, whether these alterations are due to an artifact or an abnormal heart rhythm, may diverge the attention of expert interpreters to certain hotspots in the ECG image. Davies et al [5] also concluded that experts who do not follow up the overall pattern recognition process triggered by these alterations with a formal medical systematic method for ECG interpretation are more likely to provide a wrong diagnosis. Expertise plays an important role in differentiating between waveform abnormalities that are less relevant and leads of critical importance that contribute to the accurate interpretation of the ECG, according to Wood et al [10].

Research question 3: *What is the most suitable level of granularity to analyze the eye-tracking data for the interpreter? Is it at the level of a single ECG lead or at a more granular level?*

Several studies have analyzed the eye-tracking data at the ECG lead level [6,8,10]. However, Davies et al [2] later suggested that using a more granular level of data analysis would be worth exploring, as it may enable more insight. This is especially important as the suggestion echoes Wood et al’s [10] observation that expert medical interpreters operate at both the ECG lead level and the level of the specificities of waveform abnormalities.

#### Hypothesis

Our hypothesis is that *the order with which and the duration for which medical students look at specific areas across the ECG directly affect their final interpretation*.

As commented above on research questions 1 and 2, waveform abnormalities catch the attention of the interpreter. We speculate that these abnormalities certainly catch the attention of medical students, and especially that recognizing them is at the foundation of the ECG interpretation curriculum. What then differentiates an accurate interpretation from an inaccurate one is whether students focus on the right abnormalities. This therefore influences the order with which students look at different areas of the ECG across their interpretation period, as well as how much time they spend fixating on these areas. Choosing the right data analysis tools and methodologies would be critical in obtaining accurate results to confirm or deny our hypothesis. As commented above on research question 3, finding the most suitable level of specificity to analyze the eye-tracking data for the interpreter is also part of the analysis. This is especially pertinent to understanding the dynamics of attention at the ECG lead level as well as the waveform level.

## Methods

### Materials

#### ECG Data Set Acquisition

We curated 10 ECGs of different heart arrhythmias with the assistance of a cardiology consultant. Since the experiment was designed for medical students, we made sure that these arrhythmias were all studied in traditional medical school curricula. Although some ECGs may be difficult to interpret, the students participating in the study had all the necessary medical background to make the correct diagnosis. The ECG set included in the experiment was either from the personal collection of the cardiology consultant involved in the design of the experiment or from the open ECG data set available from the PhysioNet database [11]. The 10 ECGs included are defined in Multimedia Appendix 1.

#### Experiment Design

We involved an expert in ECG interpretation as well as an expert in human-computer interaction in designing the experiment. We also referred to similar experiments described in the Related Works section. Involving these stakeholders in the design of the experiment ensured the refinement and fine-tuning of certain parameters important to lower its inherent bias. The experiment design was in the form of a digitized, short multiple-choice-question (MCQ) miniquiz, whereby medical students were exposed to the 10 ECGs on a computer screen one after the other, followed by the respective diagnosis question. This format is important as medical students are accustomed to it in their examinations. The exchange between the stakeholders involved in the design of the experiment yielded the following critical points in the experiment design:

- Randomizing the order in which the ECGs are displayed for each participant. This contributes to offsetting learning effects. This was fundamentally pertinent since (1) the task was brief (as discussed in the next point) and (2) there were many repetitions of the same task (mainly 10 repetitions of the interpretation task) [12].
- Restricting the duration for which participants are allowed to look at the ECG to 30 seconds per ECG. Deciding on this timing stemmed from the following reasons:
- The primary aim of the study was not to measure participants' performance over the accuracy of interpretation but rather to unveil visual scanning dynamics that would explain the medical students’ reasoning behind their final diagnosis provided after performing the ECG interpretation task.
- The time allowed for scanning an ECG was found to have no statistically significant effect over the accuracy of interpretation per Davies [5]. Moreover, per Bond et al [6], there was a negative correlation between the duration spent looking at an ECG and the accuracy of interpretation.
- The quality of the collected eye-tracking data: Restricting the time allowed for students to look at an ECG forces them to spend their allowed timespan wisely focusing on selected areas of the ECG. The students are therefore more attentive and wander less around the different areas of the ECG. This improves the quality of the data obtained, as will be explained more in the forthcoming sections.

#### Recruitment Process

Medical students in the classes of 2022 and 2023 enrolled in the cardiology module within the 4-year medical program curriculum were invited to participate in the study. One of the main learning objectives was the 12-lead ECG interpretation in accordance with the US medical curriculum [13]. Recruitment for this study was advertised through emails sent to both classes, as well as word-of-mouth and announcements across the students’ dormitories. Students who volunteered replied with an email to the study coordinator. Information regarding the study, including the research protocol and a consent form, was forwarded to each student following their voluntary engagement. The students were then emailed a date and time to participate in the 12-lead ECG interpretation study.

#### Task and Procedure

The students listened first to a 5-minute briefing, where they were introduced to the goal of the experiment, the number of ECGs they would interpret, and how they should interact with the interface. The interface was minimalistic in the sense that the 12-lead ECG image took up the whole computer screen and automatically changed to the next ECG after 30 seconds elapsed. The MCQ miniquiz asking about the ECG diagnosis was also minimalistic, whereby each question was centered in the screen and everything around it was grayed except the Next button to move to the next ECG. The students were allowed as much time as they needed to think about the ECG diagnosis, but in all cases, the time did not exceed 10 seconds to give their answer. The students were also encouraged to ask any question regarding the experiment or the setup. Once all the doubts were cleared, the students were set to familiarize themselves with the setup.

The students familiarized themselves with the setup by calibrating their eyes with the eye tracker's infrared sensors. Once their eyes were calibrated to the eye tracker, they proceeded to the interpretation task. The students were allowed 30 seconds to look at an ECG before being prompted for its diagnosis. Once they provided their diagnosis for the 10 ECGs, we performed an informal interview where we asked them about the overall difficulty of the interpretation task, the experiment design, and how they thought they did on the questions. Some of the students requested to check their answers.

#### Apparatus

A Tobii Pro X2-60 eye tracker and iMotions version 8.1 software (iMotions) were used to record eye movements with a sampling rate of 60 Hz (±1 Hz). Key strokes and mouse clicks were recorded to collect the students’ responses to the MCQs. The eye-tracking experiment was conducted on a 25-inch diagonal laptop monitor with a resolution of 1366 × 768 pixels.

#### Ethics

The institutional review board approval for this study was granted by the ethics board of both the Qatar Biomedical Research Institute at Hamad Bin Khalifa University [14] under the IRB Protocol Reference Number QBRI-IRB-2020-01-009 and the Hamad Medical Corporation in Qatar [15] prior to the commencement of the study. All methods were therefore performed according to the guidelines and regulations of Qatar’s Ministry of Public Health and several other international regulatory agencies [14].

### Methods

#### Heatmaps

We generate fixation heatmaps using the collected eye-tracking data as a preliminary step of data exploration. Heatmaps helped us visualize the general trends in how medical students proceed with an ECG interpretation. The observation of heatmaps contributed toward finding an answer to both research questions 1 and 2, since they indicated where medical students focus more and what they ignore on an ECG. The heatmap images of all the ECGs are available in Multimedia Appendices 2-11.

#### AOIs

**Figure 1:**
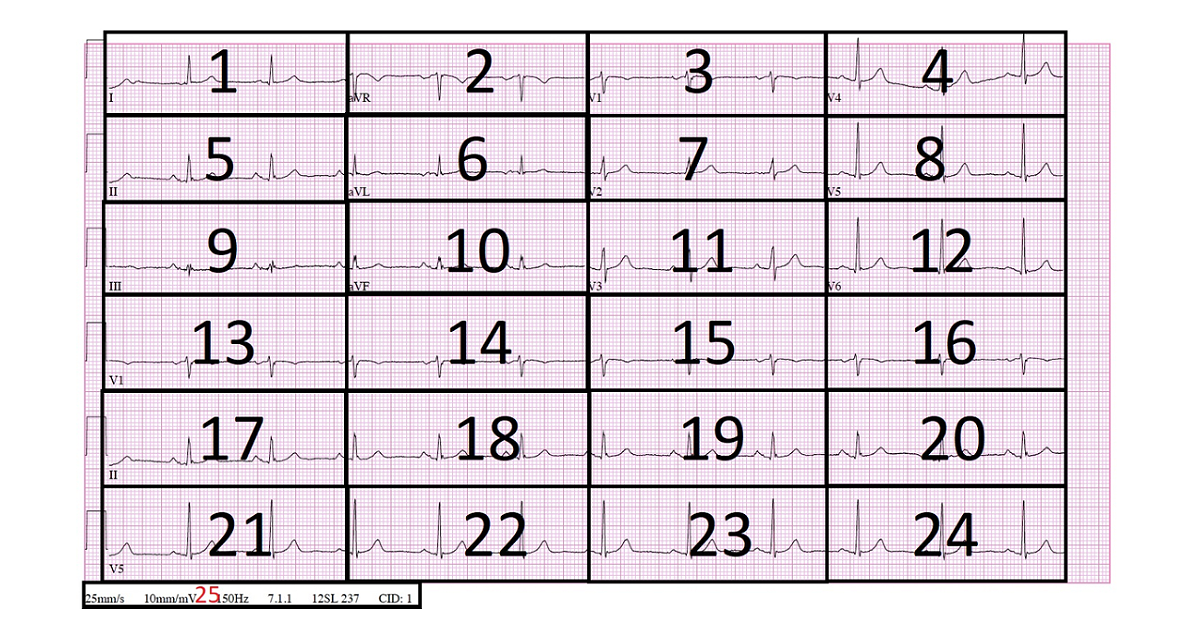
Grid-based AOI applied to the NSR ECG. AOI: area of interest; aVF: augmented vector foot; aVL: augmented vector left; aVR: augmented vector right; ECG: electrocardiogram; NSR: normal sinus rhythm.

**Figure 2:**
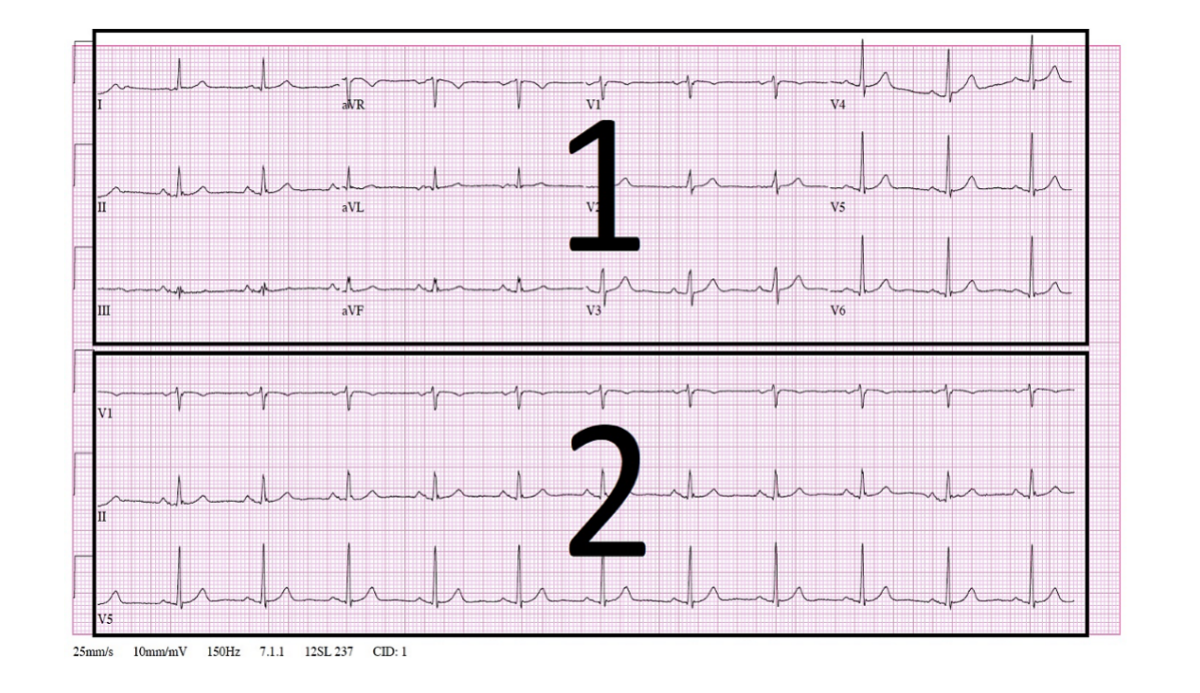
Long vs short AOI applied to the NSR ECG. AOI: area of interest; aVF: augmented vector foot; aVL: augmented vector left; aVR: augmented vector right; ECG: electrocardiogram; NSR: normal sinus rhythm.

We defined two AOI types. These two types were informed by the previous work that Davies et al [7] conducted to find the optimal bias-free AOI distribution in 12-lead ECGs (refer to a detailed AOI definition in Multimedia Appendix 12). The authors found that for AOIs to be bias free when defined on top of an ECG image, they need to be equal in area. This allows for unbiased quantification of the analyzed eye-tracking metrics. Our two defined AOI types were the long versus short AOI and the grid-based AOI. Figures 1 and 2 show the same normal sinus rhythm (NSR) ECG divided into these two different AOIs. The six horizontal lines across the ECG represent the 12 leads from where the measurements of the heart rhythm are taken. In the grid-based AOI represented in Figure 1, the ECG image is divided into 24 equal squares (AOIs) plus the scale metadata area at the bottom. Figure 2 represents the AOI of long versus short leads. This distribution divides the ECG into two equal areas. The upper area (area 1) contains the 12 leads for the ECG represented by 1 to 12 in Figure 1. These leads represent the heart voltage for a duration of 2.5 seconds (deduced from the scaling metadata at the bottom of the ECG). The lower area of the ECG represented by area 2 in Figure 2 contains three long rhythm strips for leads V1, II, and V5. The long rhythm strip represents the heart voltage of the heart activity for a duration of 10 seconds instead of 2.5 seconds. We used both of these AOI distributions in our analysis.

#### Eye Tracking and Its Features

To systematically understand how medical students proceed to interpreting an ECG, we used eye tracking. Eye tracking is a methodology that aims to quantify visual behavior when performing a specific task in order to understand the nuances in locus and levels of attention of interpreters [16]. The parameters that the eye-tracking methodology collects were suitable to test our hypothesis. The eye fixation count as well as the fixation duration were independent eye-tracking variables that referred to the “duration for which medical students look at specific areas of an ECG,” as mentioned in the hypothesis. The TTFF, as well as fixation revisitations, however, refer to the “order with which medical students look at different leads in the ECG,” as mentioned in the hypothesis. The definitions for the used eye-tracking parameters are in Multimedia Appendix 12.

#### Statistical Analysis

To measure the significance in the fixation behavior of the medical students around different AOIs on the 12-lead ECG, we use two different statistical tests. These tests were curated in accordance with the nature of the collected eye-tracking data, as well as the areas being compared.

*Comparison between the rhythm strip vs short-lead signals*: Since the comparison was between two unpaired data sets (long rhythm strip and short-lead signals) in a nonnormal distribution of data, we use the Mann-Whitney U test [17,18] on 8 ECGs out of 10. This is because the remaining 2 ECGs did not have an equal area for the selected AOIs, which made the comparison biased. The excluded ECGs were hyperkalemia and atrial fibrillation (AFib). We use an α of .05 as the cutoff for significance.

*Comparison between the 24 defined AOIs in selected ECGs*: Since we were comparing between more than two unpaired data sets (24 defined AOIs) in a nonnormal distribution of data that had the same shape, we use the Kruskal-Wallis test [19] on 8 ECGs out of 10. This is because the remaining 2 ECGs did not have an equal area for the selected AOIs, which made the comparison biased. The excluded ECGs were hyperkalemia and AFib. We use an α of .05 as the cutoff for significance.

*Correlation among eye-tracking features*: We aimed to understand the correlation between the analyzed eye-tracking metrics through the Pearson correlation coefficient. The Pearson correlation coefficient is a statistical metric that measures the strength of a linear correlation between two variables [20].

## Results

### Demographics

Table 1 summarizes the demographics for the participants in the study. In total, 16 medical students were recruited. Of those 16, 10 were in the clinical phase of their curriculum (seniors); the remaining 6 were in the preclinical phase (juniors). In addition, 15 students were male, while 1 was a female. The age of the students was between 21 and 24 years. The students were from six different countries. Eligibility for inclusion in the study was therefore mainly based on the medical program curriculum design, whereby the main condition was that students should be in their junior or senior year and have received theoretical and practical training with regard to ECG interpretation.

**Table 1:** Demographics of the eye-tracking study participants.

Feature	Demographics
Age (years)	21 (n=5); 22 (n=1); 23 (n=5); 24 (n=5)
Gender	Male: 15; female: 1
Country	Palestine (n=4); Jordan (n=6); South Korea (n=1); Egypt (n=2); Lebanon (n=2); Libya (n=1)
Class	2023 (n=6); 2022 (n=10)

### Eye-Tracking Features

We present the results for the fixation count, the time spent fixating, the average TTFF, and the average fixation revisitations on each ECG lead over all 160 interpretations (16 participants, with 10 ECGs to interpret for each student) in Table 2. The ECG Lead column is in decreasing order based on the fixation count.

**Table 2:** Results of the fixation count, the time spent fixating, the average TTFF,^a^ and the average fixation revisitations on each ECG^b^ lead for all interpreters.

ECG lead	Fixation count			Time spent fixating (ms^c^)			TTFF (ms)			Fixation revisitation count	
	Mean (μ)	SD (σ)	Mean (μ)		SD (σ)	Mean (μ)		SD (σ)	Mean (μ)		SD (σ)
II	54	9	2727		456	4225		213	2		.85
V5	28	5	1301		236	12,639		447	1		.82
V1	24	6	1476		320	9552		557	2		.73
V3	16	5	974		103	10,634		557	4		.51
V2	15	3	904		54	9898		447	4		.65
aVF^d^	14	3	890		51	6233		88	4		.62
V4 right	13	2	972		66	7035		222	3		.14
3	11	1	886		54	9222		111	3		.86
V3 right	11	1	868		47	6064		93	3		.37
2	11	1	1017		199	8683		38	4		.47
aVL^e^	10	1	723		47	3602		103	3		.82
V7	8	1	437		48	10,513		601	2		.92
1	8	1	707		42	11,393		587	2		.80
V8	8	1	435		53	13,076		598	1		.51
aVR^f^	8	1	456		49	11,569		615	1		.85
V6	7	1	325		49	18,641		566	1		.59
V4	6	1	312		32	19,460		333	1		.20
Info (metadata)	4	1	64		16	20,394		887	0		.57

^a^TTFF: time to first fixation.

^b^ECG: electrocardiogram.

^c^ms: milliseconds.

^d^aVF: augmented vector foot.

^e^aVL: augmented vector left.

^f^aVR: augmented vector right.

### Medical Students’ Interpretation Results

**Figure 3:**
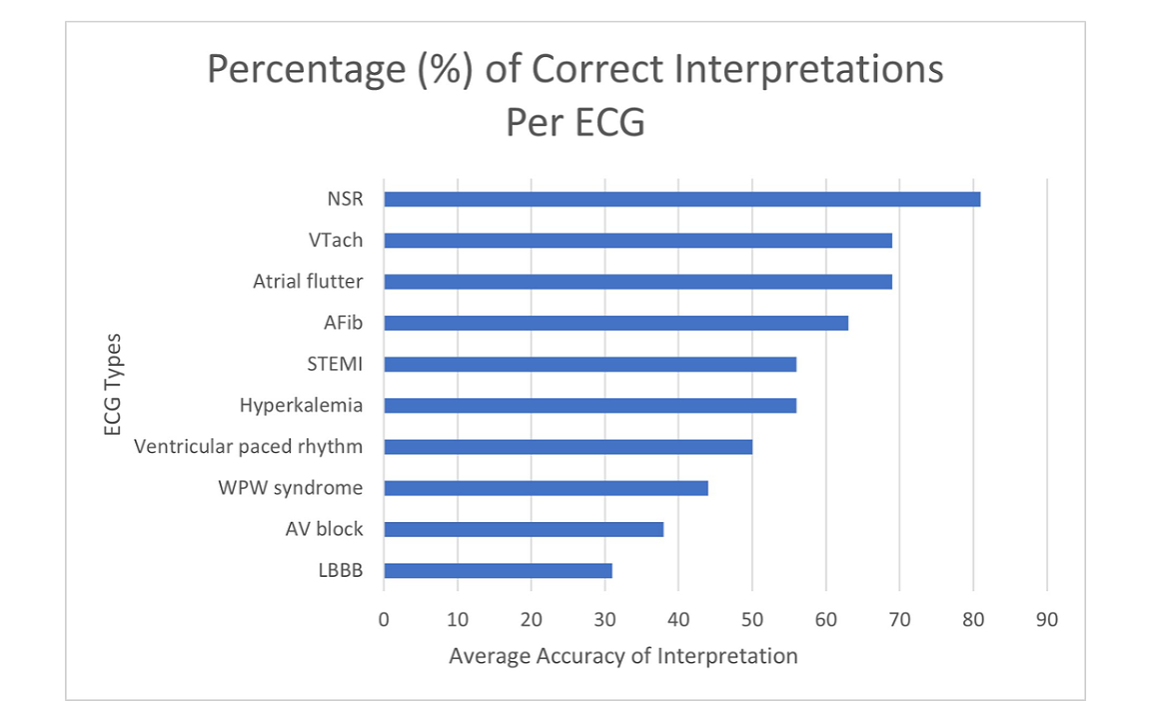
Percentage (%) of correct interpretations per ECG for all medical students. AFib: atrial fibrillation; AV block: complete heart block; ECG: electrocardiogram; LBBB: left bundle branch block; NSR: normal sinus rhythm; STEMI: ST-segment elevation myocardial infarction; VTach: ventricular tachycardia; WPW: Wolf-Parkinson-White.

Figure 3 shows the percentage of correct interpretations per ECG for all the medical students. Multimedia Appendix 13 summarizes the results of medical students’ interpretations in more detail. The mean overall score of correct ECG answers was 55.63%, and the SD was 4.63%. The NSR was the ECG with the highest number of correct interpretations with an accuracy of 81%, while the left bundle branch block (LBBB) was the ECG with the lowest number of correct interpretations with an accuracy of 31%.

### Data Analysis

We analyzed the collected eye-tracking data. We started by defining key parameters on which the data analysis was founded, mainly AOIs. This contributed to answering research question 3. We then proceeded to find an answer to research question 1 by analyzing the eye-tracking parameters. These parameters are referred to in the hypothesis as the “duration for which medical students look at specific areas of the ECG.” They are the fixation count and the fixation duration. “Specific areas of the ECG,” as mentioned in the hypothesis therefore refer to the defined AOIs. We finally analyzed the correlation between these four eye-tracking features to test our hypothesis.

#### Selected Features

We selected the fixation count and the fixation duration as the main features guiding our analysis to answer research question 1.

**Figure 4:**
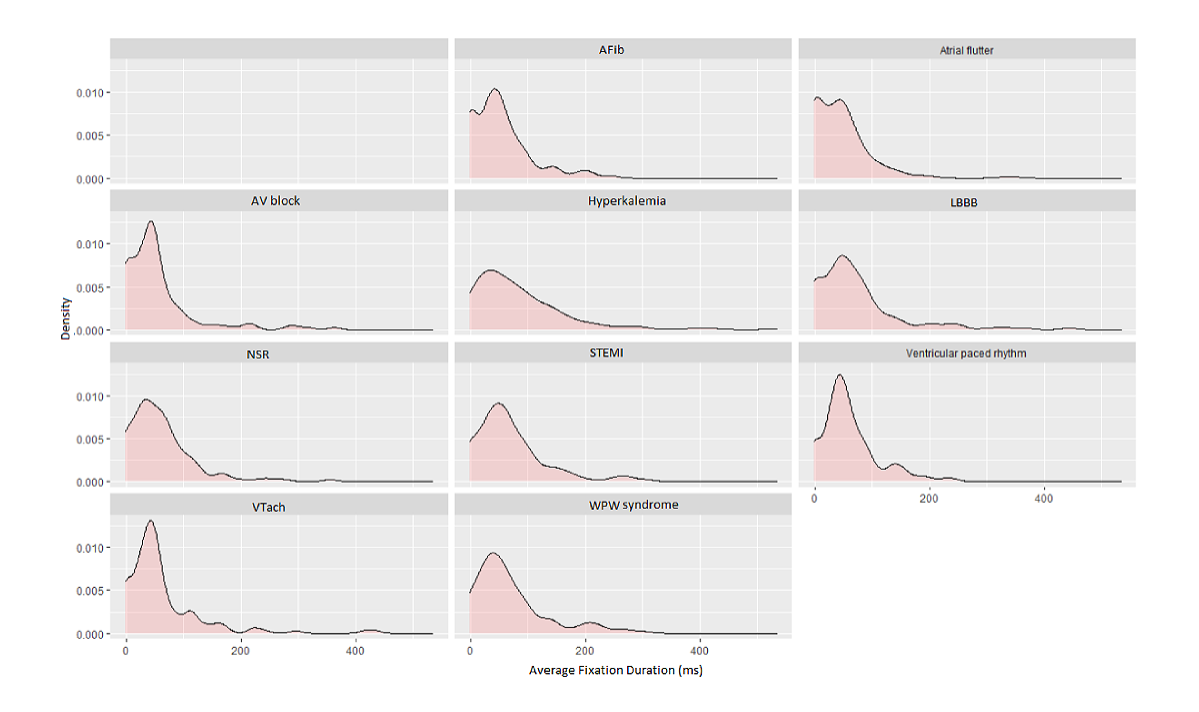
Density distribution of the average fixation duration for all ECG interpreters and all ECGs. AFib: atrial fibrillation; AV block: complete heart block; ECG: electrocardiogram; LBBB: left bundle branch block; ms: milliseconds; NSR: normal sinus rhythm; STEMI: ST-segment elevation myocardial infarction; VTach: ventricular tachycardia; WPW: Wolf-Parkinson-White.

*Fixation duration*: Figure 4 displays the density distribution of the average fixation duration for all ECG interpreters across each ECG. The distribution was left-skewed, centered at around 100 ms for all the ECGs. Moreover, some of the ECGs had a bi-modal distribution (like the atrial flutter distribution). The reason for these characteristics is the diversity of the fixation distribution across each defined AOI in the grid-based AOI distribution shown in Figure 1 and in Table 2. Additionally, for the ECGs that had a bimodal distribution, some of the peaks in the distribution graph were centered around the 0 ms value. This might be due to some AOIs in the ECG not being visited at all across the duration of the interpretation.

*Fixation count*: We conducted statistical tests on the fixation count according to both defined AOIs in Figures 1 and 2. This was with the aim to better elucidate how significant the difference is in terms of fixation counts (1) between the rhythm strips versus the short signal timing leads and (2) within all the 24 AOIs in the ECG. Analyzing the fixation count according to those two defined AOIs contributed to providing an answer to research question 3.

(1) Between the rhythm strip vs the short-lead signals: We used the Mann-Whitney U test, as discussed in the Statistical Analysis section. As observed in Table 3, there were only two ECGs that had a *P* value less than the cutoff for significance. These ECGs were the NSR and atrial flutter ECGs. When we looked at the mean fixation count in the rhythm strips versus the mean in the normal leads, we observed the significance of the difference, whereby the mean fixation count over the rhythm strips was significantly larger than the mean in the normal leads. For the remaining ECGs, the mean fixation count was not significantly different between both AOIs. Translating this to eye-tracking behavior, medical students usually refer to all the leads, whether they are rhythm strips or only short 2.5-second leads. For the two cases mentioned, the medical students referred mostly to the long rhythm strips to reach to a diagnosis. For the remaining ECGs, the interpreters had to observe and analyze both long and short leads relatively equally in time in order to reach a diagnosis.

(2) Between the 24 defined AOIs in selected ECGs: We used the Kruskal-Wallis test, as discussed in the Statistical Analysis section. The results from applying the test indicated that all the ECGs had a *P* value significantly less than the cutoff for significance (<.001). Translating this to eye-tracking behavior, at the granular level, there was a significant difference in the proportion of fixations that the medical students interpreting the ECGs accorded to different AOIs. This means that the students’ focus on different AOIs varied according to the ECG.

**Table 3:** Mann-Whitney U test conducted on the fixation count parameter of selected ECGs.^a^

ECG	*P* value	Mean fixation count in rhythm strips	Mean fixation count in shorter strips
NSR^b^	.010*	169.875	98.750
Atrial flutter	.015*	182.625	54.125
VTach^c^	1	118.000	131.875
WPW^d^	.958	144.500	145.625
Ventricular paced rhythm	.279	126.375	162.250
LBBB^e^	.161	143.250	115.75
STEMI^f^	.721	119.25	125.25
AV block^g^	.248	157.875	117.875

*Values in italic are significant.

^a^ECG: electrocardiogram.

^b^NSR: normal sinus rhythm.

^c^VTach: ventricular tachycardia.

^d^WPW: Wolf-Parkinson-White.

^e^LBBB: left bundle branch block.

^f^STEMI: ST-segment elevation myocardial infarction.

^g^AV block: complete heart block.

#### Correlation Among Eye-Tracking Features

We used the Pearson correlation coefficient, as discussed in the Materials and Methods section. Table 4 reports the coefficients among the selected eye-tracking features using the grid-based AOI described in Figure 1.

**Table 4:** Pearson correlation coefficient for eye-tracking parameters over the 10 studied ECGs.^a^

	Fixation count	Fixation duration	TTFF^b^	AOI^c^ fixation revisitations
Fixation count	—^d^	.81	–.4	.78
Fixation duration	—	—	–.36	.53
TTFF	—	—	—	–.44
AOI fixation revisitations	—	—	—	—

^a^ECG: electrocardiogram.

^b^TTFF: time to first fixation.

^c^AOI: area of interest.

^d^Not applicable.

Table 4 indicates that the fixation count as well as the fixation duration reflect the same ECG interpretation behavior as they are strongly correlated. The remaining combinations of features indicated that each variable represents a distinct ECG interpretation behavior as the variables are weakly correlated.

## Discussion

Here, we discuss the Results section. We mainly discuss the insights from the heatmaps as well as the analysis of the eye-tracking features.

### Heatmaps

As observed in all the heatmaps, medical students tend to lean toward one of two distinct behaviors for ECG interpretation. The nuance lies primarily in the areas where the fixations are concentrated.

1. In some ECGs, such as the NSR and the atrial flutter, eye fixations are concentrated over the lower area. This area contains the three leads (V1, II, and V5) referred to as rhythm strips.

2. In other ECGs, such as the ventricular paced rhythm, eye fixations are concentrated in the upper half, precisely over leads V1, V2, and V3.

The generated heatmaps indicate how complex and different the ECG interpretation approach is depending on the heart pathology observed. They also indicate that medical students adapt their interpretation behavior, depending on the nature of the ECG. The preliminary observations from the heatmaps show that medical students fixate more on abnormal areas of the ECG, such as atrial flutter waves, wide QRSs, elevated ST segments, and V-pacing spikes, regardless of whether their interpretation of the ECG is correct. These observations provide an answer to research question 2.

### Eye-Tracking Features

The eye-tracking features reported in the Results section and analyzed previously confirm the observations made when looking at heatmaps. The fixation count as well as the average fixation duration features demonstrate that medical students fixate the most on rhythm strips, mainly lead II, followed by leads V1 and V5. This might be because rhythm strips provide the best longitudinal analysis of the rate and rhythm over the entirety of the ECG. According to the TTFF and the fixation revisitations per lead, medical students’ natural tendency is to start their interpretation from the middle of the ECG. They then move to whatever lead they believe guides them to a correct interpretation. Specificities of their interpretation changes from one ECG to the other. Amidst the interpretation process, waveform abnormalities may catch their attention and divert their attention from one lead to another, provoking an increase in lead revisitations. These observations answer research question 1.

### Answers to Research Questions

Research question 1: We recognized two main eye-tracking patterns that medical students often repeat during the process of ECG interpretation. The first pattern is that they start from the leads in the center of the ECG. The second pattern is that across the interpretation period, they refer primarily to the rhythm strips as the guiding leads toward finding an indicator for the appropriate diagnosis, as they spend most of the interpretation time fixating on these rhythm strips. This behavior of interpretation was also found by Davies et al [5], who defined this pattern as a systematic way of interpretation. By “a systematic methodology of interpretation,” the authors referred to a set of routinely repeated observations across all ECG types [5]. In a purely systematic way of interpretation, the interpreter examines the ECG thoroughly.

Research question 2: The presence of one or multiple abnormal waveforms in the ECG does affect the ECG interpretation behavior of medical students. The abnormalities may be due to an actual heart rhythm irregularity or due to noise. These abnormalities usually derail the students from a systematic approach of interpretation. The abnormal signs catch their attention and therefore make them fixate longer and often switch between leads where these abnormalities occur. Davies et al [5] defined this behavior of interpretation as a “reactive” one. Although this behavior was found among expert interpreters, it was found on rare occasions as opposed to our findings. A reactive way of interpretation refers to a methodology where the interpretation is driven by abnormalities in the ECG that catch the attention of the interpreter. Experts selectively react to these signs as opposed to beginner interpreters.

Research question 3: We could not identify one suitable level of specificity to analyze the eye-tracking data. This is because the ECGs’ complexity of interpretation varies depending on the heart abnormality being tracked. However, the statistical analysis that we conducted offered an understanding of how to optimize AOIs to get meaning out of the eye-tracking data. The level of granularity varies between comparing rhythm strips against short-lead signals at one end of the spectrum. On the other end of the spectrum, the comparison is done by dividing the whole ECG into equal grid sizes in order to compare the eye-tracking feature among the grids. The challenge with grid-based analysis is that it is extremely granular and that the difference between grids is usually always significant. Grid-based analysis is suitable for understanding the-eye tracking behavior around specific waveform abnormalities.

### Hypothesis Confirmation

We confirmed our hypothesis that the order with which and the duration for which medical students look at specific areas across the ECG directly affect their final interpretation. This was demonstrated through the distribution of the fixation count as well as the fixation duration across different AOIs over the ECG. Additionally, the TTFF as well as the AOI fixation revisitations proved that some ECG leads catch the attention of interpreters more than others. This is especially true when the lead contains an abnormal signal.

### Limitations

A major limitation that affected our work was the confounding effect of a common training framework for all participants. This was because all the participants recruited to the study received the same ECG interpretation training. This effect needs to be acknowledged as influencing the results, and therefore addressed. The study conducted by Breen et al [4] could be used in our case as a starting point to address this limitation. The study provides a different perspective of how medical students with different educational backgrounds proceed to interpreting ECGs. Breen et al [4] found that the average time taken by medical students to interpret an ECG ranges between 13.2 and 59.5 seconds for similar ECG cases. This behavior supports our decision to limit the maximum time allowed for a student to look at an ECG to 30 seconds. Breen et al [4] also found that “the rhythm strip is the most common lead studied and fixated on for the longest duration, while lead I is studied for the shortest duration.” These findings by Breen et al [4] echo the results of our experiment. Although the major findings of Breen et al [4] support our findings, the most appropriate way to address this limitation is to increase the sample size and diversify it by looking for participants with different educational backgrounds and different demographics in general.

### Future Work

The reported results are considered the first phase of a larger study. In future works, we plan on expanding the study by directly addressing the limitations mentioned in the previous section. We aim to increase the number of participants to be more diverse in terms of their educational background, demographics, and expertise in ECG interpretation. We aim to include more medical students, nurses, technicians, cardiology fellows, residents, and consultants. This increase in the number of participants will enable the examination of nuances in interpretation behavior across the whole medical practitioners’ spectrum. The prospects of this participant expansion include the ability to compare the results against related studies. This data will contribute to the development of a more detailed road map of how health practitioners proceed to interpreting ECGs.

### Conclusion

We presented a quantitative analysis of the ECG interpretation behavior of medical students. We collected eye-tracking data of 16 medical students interpreting 10 ECGs each. This enabled us to analyze these data using a number of different eye-tracking features, mainly AOIs, fixation count, fixation duration, TTFF, and fixation revisitations. This was with the aim of answering three research questions: (1) whether we can recognize any interpretation patterns within the medical students’ population, (2) what elements in the ECG catch the attention of medical students during their interpretation process, and (3) and, finally, what the most suitable level of granularity is to analyze the eye-tracking data for optimal insights. We found that medical students often start their interpretation by fixating at the center of the ECG. This may be due to the central bias tendency toward fixating at the center of the observed image. This bias is widely observed in eye-tracking research, as reported by Bindemann et al [21]. However, in some other cases, medical students focus on the central leads to find any abnormalities in the ECG waveform. Abnormalities divert their attention, which pushes them to transition across different leads. However, rhythm strips remain their guiding point toward identifying indicators, leading to the correct interpretation. These findings confirm our hypothesis that the order with which and the duration for which medical students look at specific areas across the ECG directly affect their final interpretation.

## Appendix

Multimedia Appendix 1 Electrocardiograms and their definitions.

Multimedia Appendix 2 Heatmap for a Wolf-Parkinson-White syndrome electrocardiogram interpreted by medical students.

Multimedia Appendix 3 Heatmap for a ventricular tachycardia electrocardiogram interpreted by medical students.

Multimedia Appendix 4 Heatmap for a ventricular paced rhythm electrocardiogram interpreted by medical students.

Multimedia Appendix 5 Heatmap for an ST-segment elevation myocardial infarction electrocardiogram interpreted by medical students.

Multimedia Appendix 6 Heatmap for a normal sinus rhythm electrocardiogram interpreted by medical students.

Multimedia Appendix 7 Heatmap for a left bundle branch block electrocardiogram interpreted by medical students.

Multimedia Appendix 8 Heatmap for a hyperkalemia electrocardiogram interpreted by medical students.

Multimedia Appendix 9 Heatmap for a complete heart block electrocardiogram interpreted by medical students.

Multimedia Appendix 10 Heatmap for an atrial flutter electrocardiogram interpreted by medical students.

Multimedia Appendix 11 Heatmap for an atrial fibrillation electrocardiogram interpreted by medical students.

Multimedia Appendix 12 Eye-tracking parameter definitions.

Multimedia Appendix 13 Interpretation answers by medical students.

## Abbreviations

AFib: atrial fibrillation
AOI: area of interest
AV block: complete heart block
aVF: augmented vector foot
aVL: augmented vector left
AVNRT: atrioventricular nodal reentry tachycardia
aVR: augmented vector right
ECG: electrocardiogram
LBBB: left bundle branch block
MCQ: multiple-choice question
NSR: normal sinus rhythm
STEMI: ST-segment elevation myocardial infarction
TTFF: time to first fixation
VTach: ventricular tachycardia
WPW: Wolf-Parkinson-White
