# Supplementary material for: Interpretation of a 12-Lead Electrocardiogram by Medical Students: Quantitative Eye-Tracking Approach
Source: JMIR Med Educ. 2021 Oct 14;7(4):e26675. doi: 10.2196/26675 (PMC8554676; doi:10.2196/26675)
Supplement: Multimedia Appendix 1 [file mededu_v7i4e26675_app1.pdf]

Multimedia Appendix 1: ECGs[a] and their definitions.

ECG	Abbreviation	Definition
Normal sinus rhythm	NSR	A normal ECG rhythm.
Atrial fibrillation	AFib	Irregular, often rapid heart rate that commonly causes poor blood flow.
Hyperkalemia	NA[b]	Potassium level imbalance in the blood that is higher than normal.
Atrial flutter	NA	An arrhythmia causing a rapid heart rate.
Ventricular tachycardia	VTach	Fast ventricular rate.
Wolff-Parkinson-White syndrome	WPW syndrome	An accessory pathway between the atria and ventricles.
Ventricular paced rhythm	NA	An artificial pacemaker inserted to activate the ventricle(s).
Left bundle branch block	LBBB	Blockage of electrical impulse traveling down the left bundle branch.
ST-segment elevation myocardial infarction	STEMI	A heart attack during which one of the heart's major arteries is blocked. There are different locations of STEMI that look different on the ECG: anterior STEMI, inferior STEMI, lateral STEMI, anterolateral STEMI, and inferolateral STEMI.
Complete heart block	AV block	A medical condition in which the nerve impulse generated in the sinoatrial node in the atrium of the heart cannot propagate to the ventricles.

[a]ECG: electrocardiogram

[b]NA: not available.
