# Supplementary figures and images for: Interpretation of a 12-Lead Electrocardiogram by Medical Students: Quantitative Eye-Tracking Approach
Source: JMIR Med Educ. 2021 Oct 14;7(4):e26675. doi: 10.2196/26675 (PMC8554676; doi:10.2196/26675)

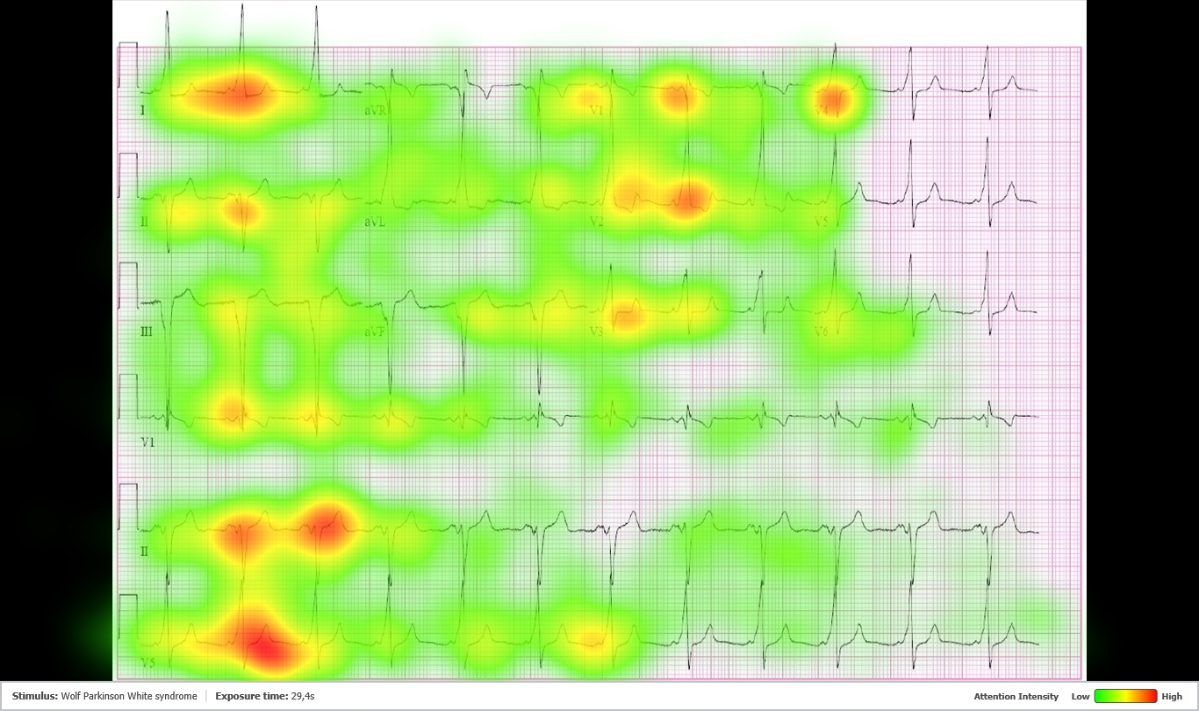

Supplement: Multimedia Appendix 2 [file mededu_v7i4e26675_app2.png]

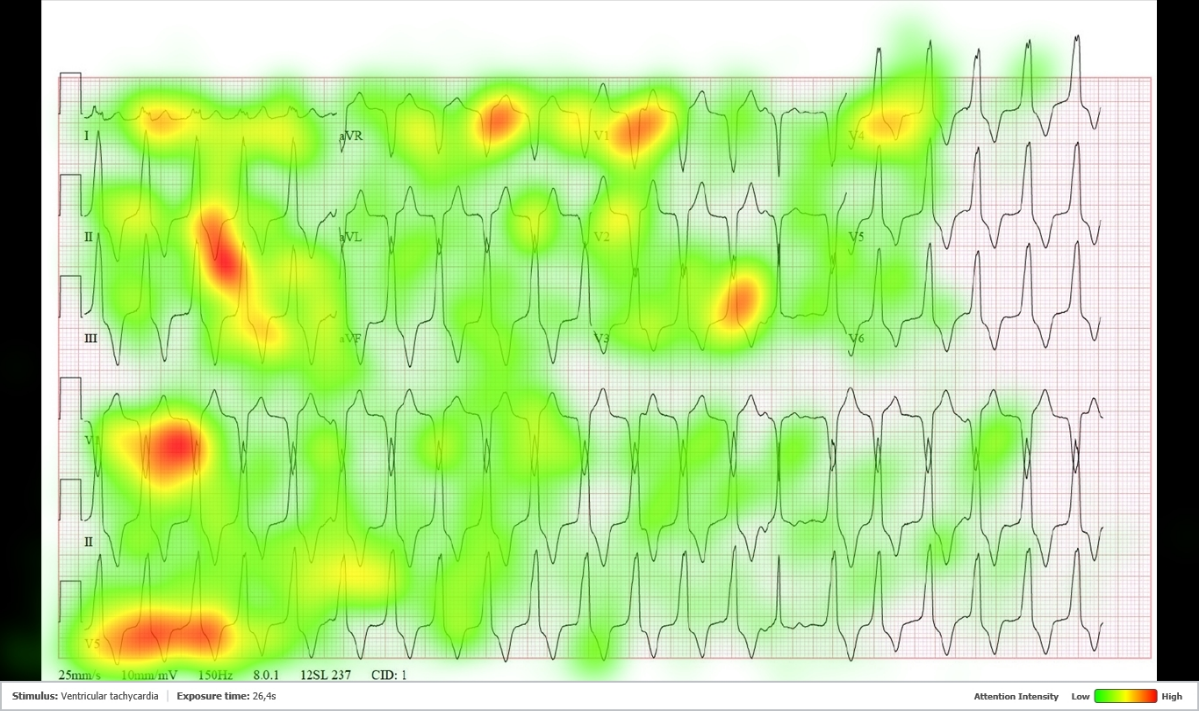

Supplement: Multimedia Appendix 3 [file mededu_v7i4e26675_app3.png]

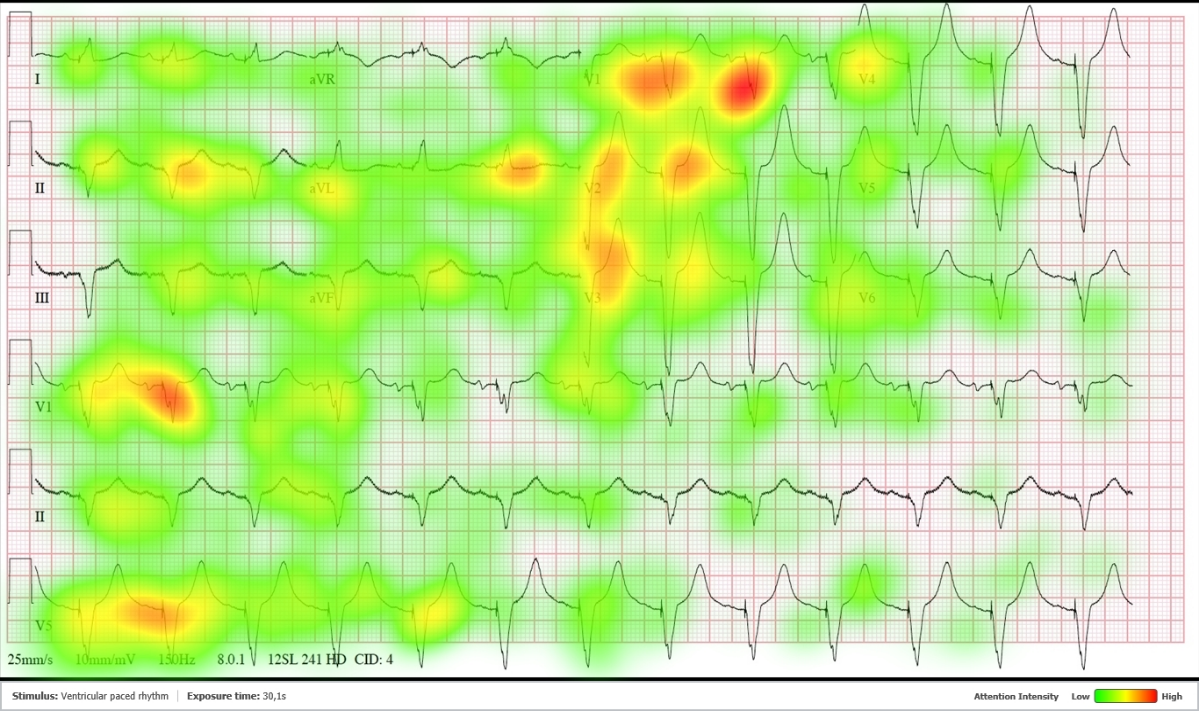

Supplement: Multimedia Appendix 4 [file mededu_v7i4e26675_app4.png]

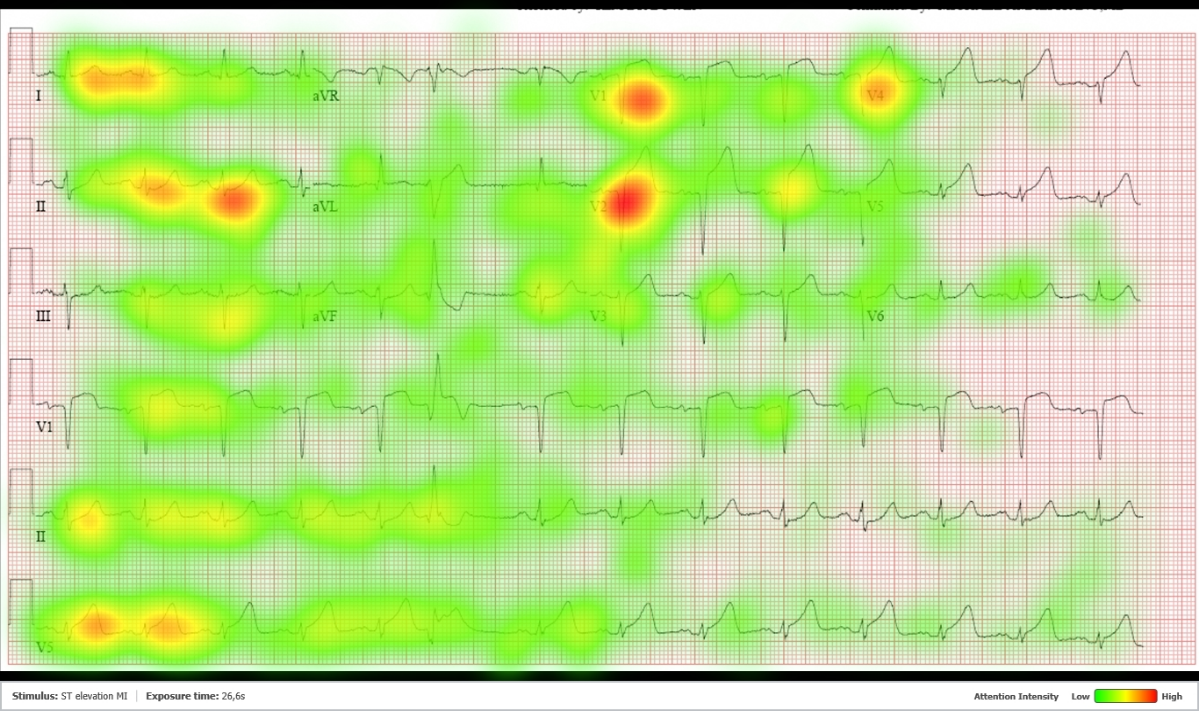

Supplement: Multimedia Appendix 5 [file mededu_v7i4e26675_app5.png]

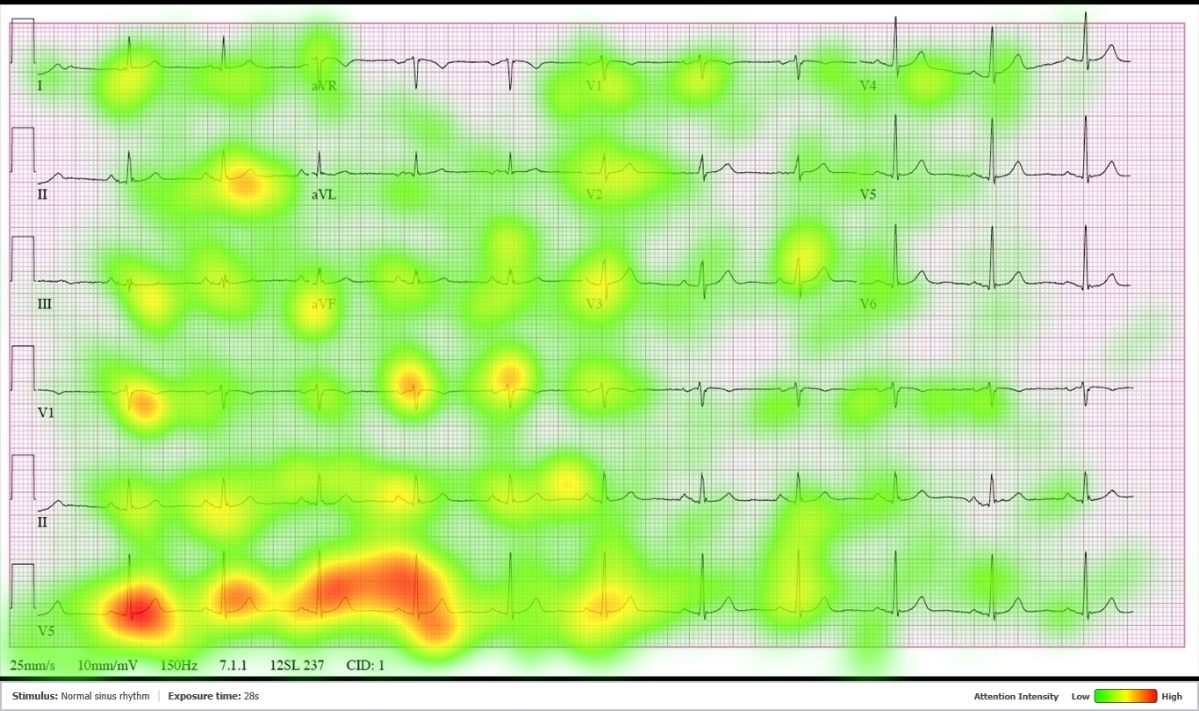

Supplement: Multimedia Appendix 6 [file mededu_v7i4e26675_app6.png]

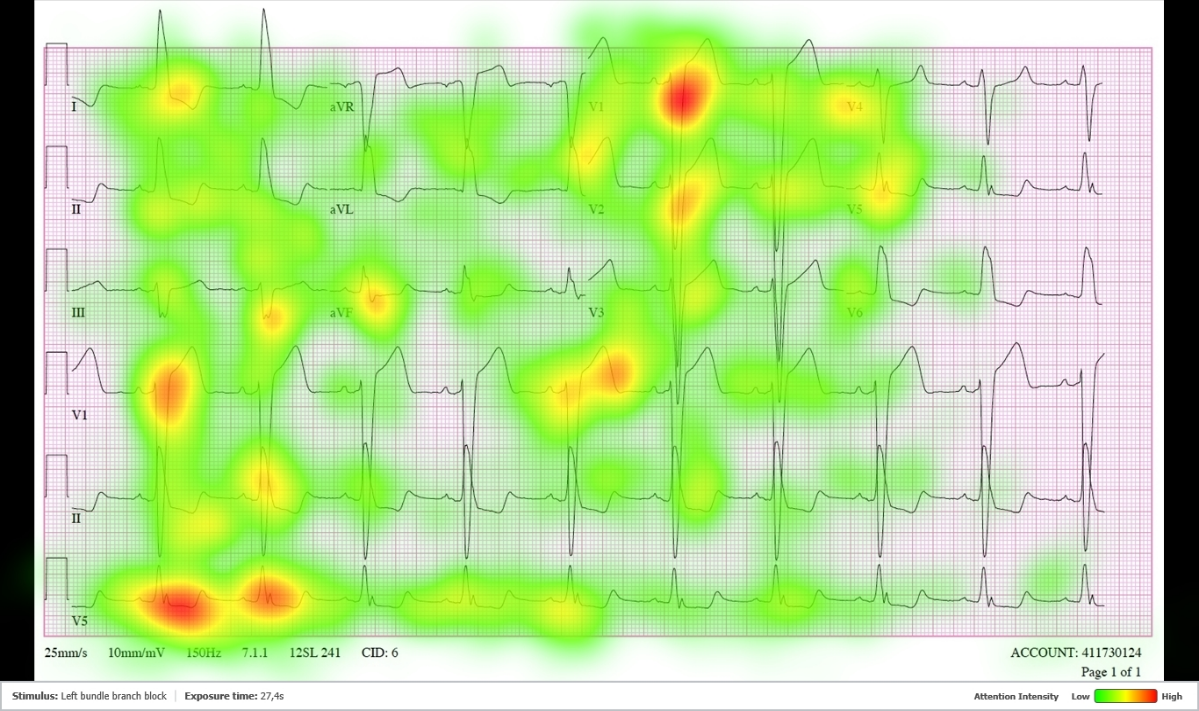

Supplement: Multimedia Appendix 7 [file mededu_v7i4e26675_app7.png]

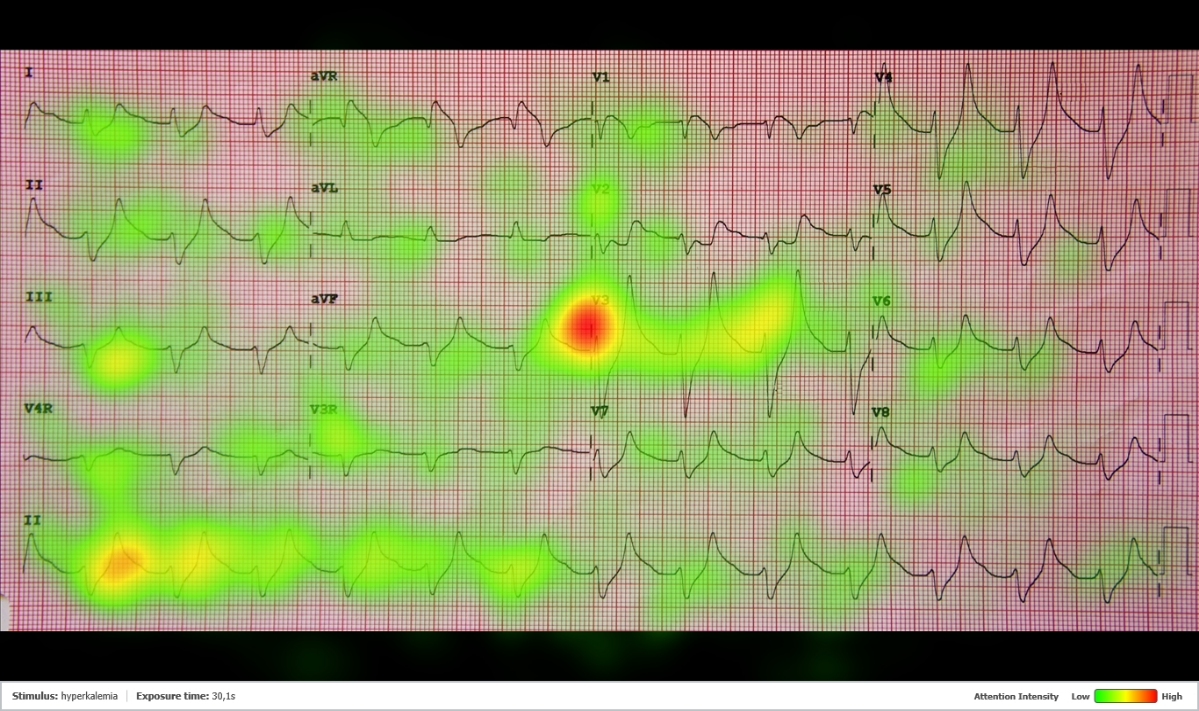

Supplement: Multimedia Appendix 8 [file mededu_v7i4e26675_app8.png]

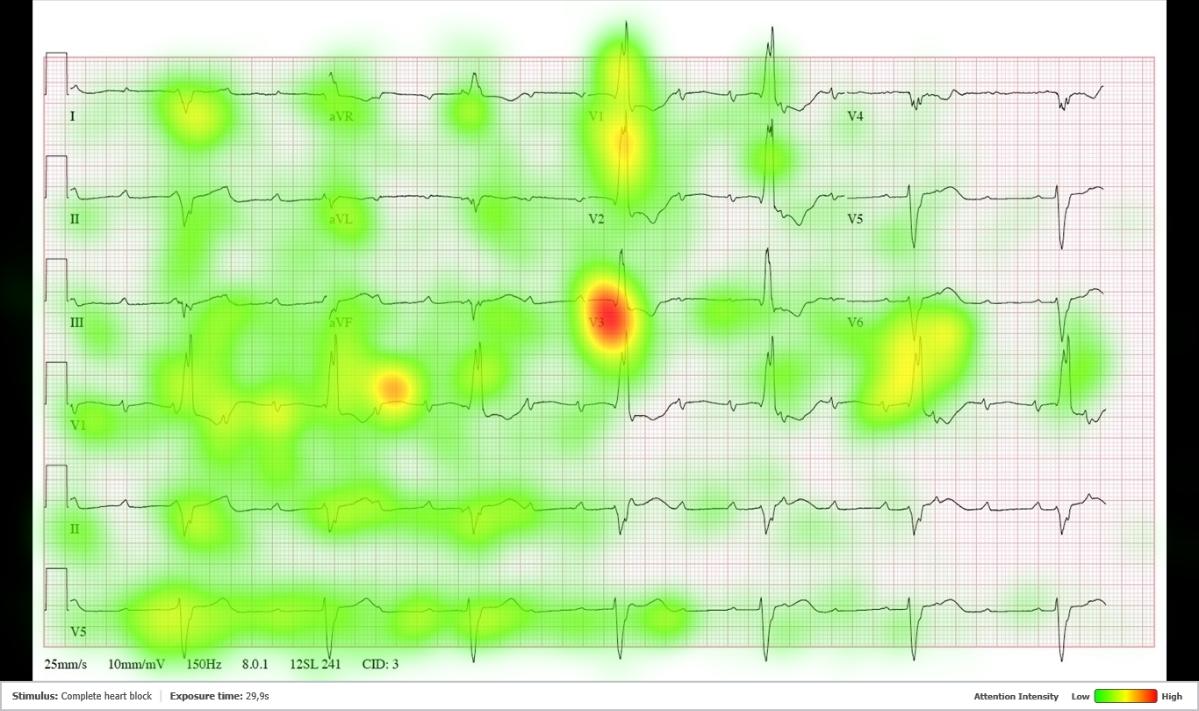

Supplement: Multimedia Appendix 9 [file mededu_v7i4e26675_app9.png]

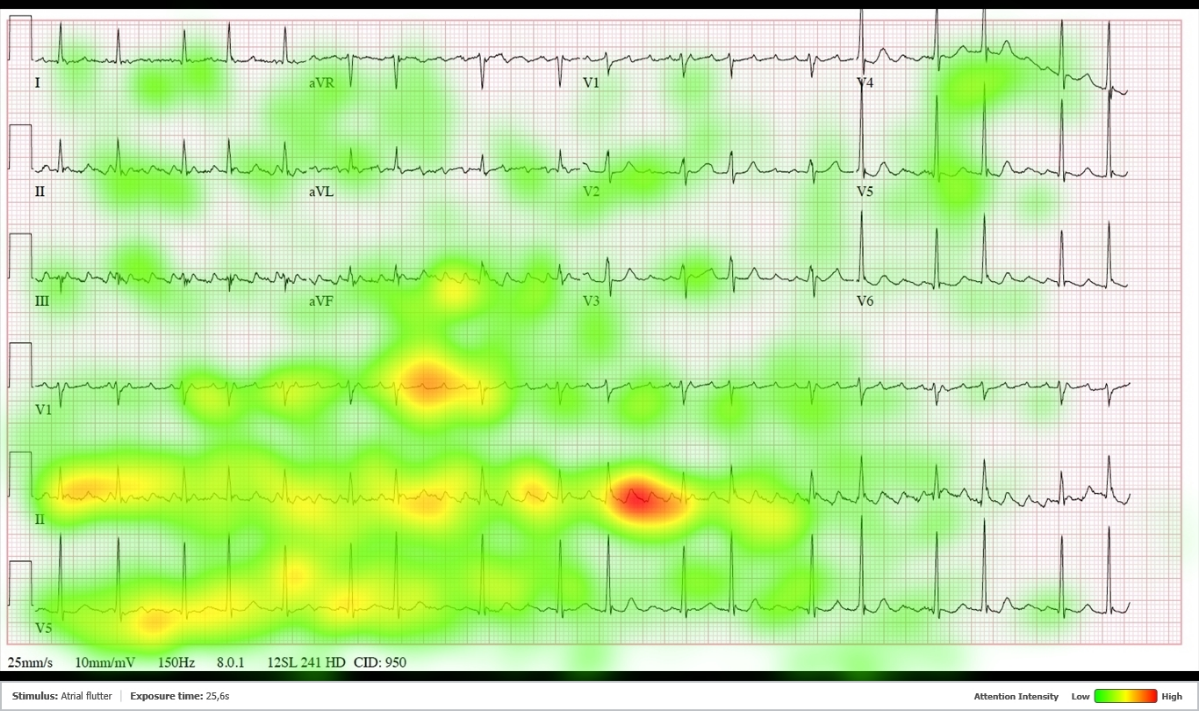

Supplement: Multimedia Appendix 10 [file mededu_v7i4e26675_app10.png]

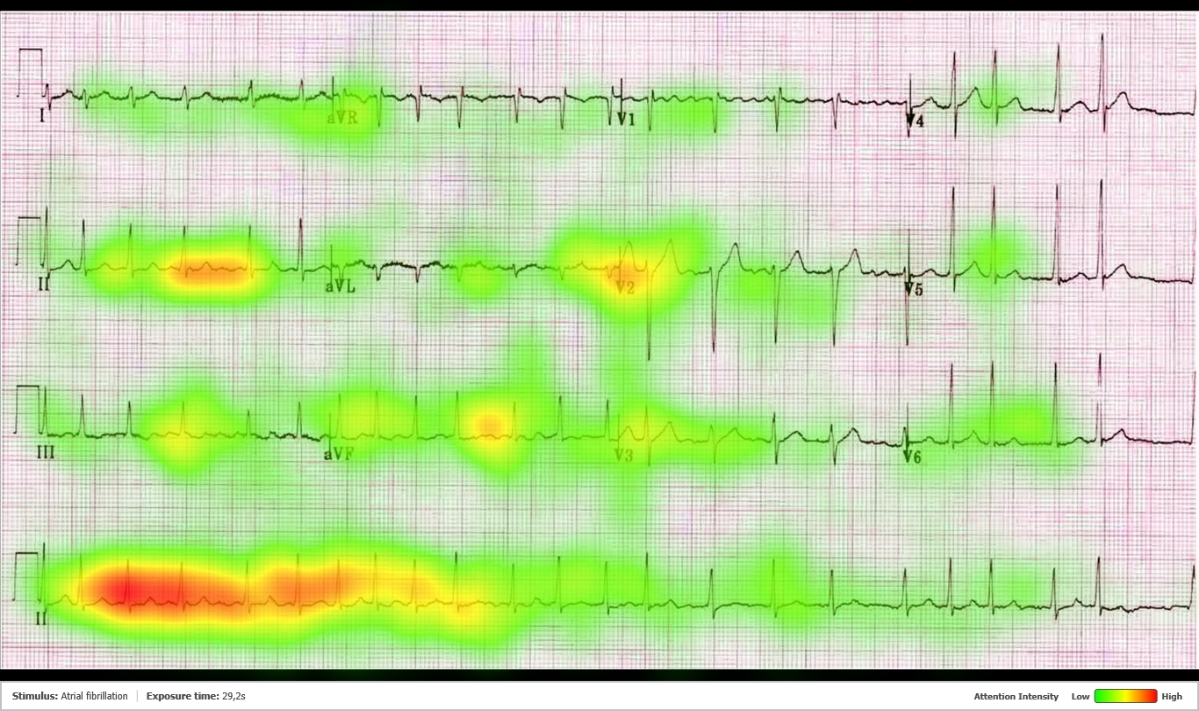

Supplement: Multimedia Appendix 11 [file mededu_v7i4e26675_app11.png]
