# Supplementary material for: Interpretation of a 12-Lead Electrocardiogram by Medical Students: Quantitative Eye-Tracking Approach
Source: JMIR Med Educ. 2021 Oct 14;7(4):e26675. doi: 10.2196/26675 (PMC8554676; doi:10.2196/26675)
Supplement: Multimedia Appendix 12 [file mededu_v7i4e26675_app12.pdf]

Multimedia Appendix 12: Eye-tracking parameter definitions.

Parameter	Definition
AOIs[a]	AOIs are an eye-tracking tool for selecting regions of a displayed stimulus. This enables the extraction of metrics specifically for those regions. Although not strictly a metric by itself, the AOI defines the area by which other metrics are calculated. AOIs allow for the quantification of visual attention at both the aggregate and the individual level [22].
TTFF[b] (ms)	Time stamp of the first fixation inside the AOI.
Fixation duration (ms)	Time spent in the AOI, based on the total duration of all the respondent's fixations (excludes data points between fixations).
Fixation revisitations	Average number of returns to the AOI.
Fixation count	Number of fixations recorded inside the AOI.

[a]AOI: area of interest.
[b]TTFF: time to first fixation.
