# Supplementary material for: Interpretation of a 12-Lead Electrocardiogram by Medical Students: Quantitative Eye-Tracking Approach
Source: JMIR Med Educ. 2021 Oct 14;7(4):e26675. doi: 10.2196/26675 (PMC8554676; doi:10.2196/26675)
Supplement: Multimedia Appendix 13 [file mededu_v7i4e26675_app13.pdf]

Multimedia Appendix 13: Interpretation answers by medical students.

Multimedia Appendix 13 summarizes the results of medical students' interpretations. In the table, *n* refers to the number of students who chose one of the four proposed choices on the electrocardiogram (ECG) experiment. The Other column refers to any other ECG interpretation the students gave other than the four provided choices. The correct choice, according to our expert involved in the design of the experiment, is the one in the first column. The percentage in the first column refers to the percentage of students who gave a correct interpretation of the mentioned ECG.

ECG[a]	Choice 1	Choice 2	Choice 3	Choice 4	Other
NSR[b] 81.25%	NSR (n = 13)	VTach[c] (n = 0)	AFib[d] (n = 1)	Atrial tachycardia (n = 1)	Sinus bradycardia (n = 1)
AFib 62.5%	NSR (n = 1)	VTach (n = 2)	AFib (n = 10)	Atrial flutter (n = 2)	Wenckebach (n = 1)
Hyperkalemia 56.25%	STEMI[e] (n = 2)	Hyperkalemia (n = 9)	VTach (n = 3)	Atrial flutter (n = 4)	AVNRT[f] (n = 1)
Atrial flutter 68.75%	WPW[g] syndrome (n = 1)	VTach (n = 0)	AFib (n = 3)	Atrial flutter (n = 11)	AV block[h] (n = 1)
VTach 68.75%	Ventricular fibrillation (n =4)	VTach (n = 11)	NSR (n =0)	Atrial tachycardia (n = 1)	NA[i]
WPW syndrome 43.75%	Brugada syndrome (n = 4)	WPW syndrome (n = 7)	Long QT syndrome (n = 2)	NSR (n = 3)	NA
Ventricular paced rhythm 50%	Ventricular paced rhythm (n = 8)	RBBB[j] (n = 3)	Atrial paced rhythm (n = 1)	Hypercalcemia (n = 3)	LBBB[k] (n = 1)
LBBB 31.25%	Ventricular paced rhythm (n = 2)	RBBB (n = 5)	VTach (n = 0)	LBBB (n = 5)	STEMI (n = 1)<br><br>Bundle branch block (unclear) (n = 1)

STEMI 56.25%	Ventricular paced rhythm (n = 1)	Non-STEMI (n = 2)	STEMI (n = 9)	Acute pericarditis (n = 1)	No answer (n = 1)<br><br>Heart block type 1 (n =1)<br><br>Left AV block (n = 1)
AV block 37.5%	Sinus bradycardia (n = 4)	First-degree AV block (n = 6)	AV block (n = 6)	VTach	NA

[a]ECG: electrocardiogram.

[b]NSR: normal sinus rhythm.

[c]VTach: ventricular tachycardia.

[d]AFib: atrial fibrillation.

[e]STEMI: ST-segment elevation myocardial infarction.

[f]AVNRT: atrioventricular nodal reentry tachycardia.

[g]WPW: Wolf-Parkinson-White.

[h]AV block: complete heart block.

[i]NA: not available.

[j]RBBB: right bundle branch block.

[k]LBBB: left bundle branch block.
